# There and Back Again: Leptin Actions in White Adipose Tissue

**DOI:** 10.3390/ijms21176039

**Published:** 2020-08-21

**Authors:** Noelia Martínez-Sánchez

**Affiliations:** Department of Physiology, Anatomy and Genetics, University of Oxford, Oxford OX1 3PT, UK; noelia.martinezsanchez@dpag.ox.ac.uk

**Keywords:** leptin, white adipose tissue, obesity

## Abstract

Leptin is a hormone discovered almost 30 years ago with important implications in metabolism. It is primarily produced by white adipose tissue (WAT) in proportion to the amount of fat. The discovery of leptin was a turning point for two principle reasons: on one hand, it generated promising expectations for the treatment of the obesity, and on the other, it changed the classical concept that white adipose tissue was simply an inert storage organ. Thus, adipocytes in WAT produce the majority of leptin and, although its primary role is the regulation of fat stores by controlling lipolysis and lipogenesis, this hormone also has implications in other physiological processes within WAT, such as apoptosis, browning and inflammation. Although a massive number of questions related to leptin actions have been answered, the necessity for further clarification facilitates constantly renewing interest in this hormone and its pathways. In this review, leptin actions in white adipose tissue will be summarized in the context of obesity.

## 1. Introduction

Overweightness and obesity are medical conditions that are rising globally in which excess body fat is accumulated. Among the various health effects, this condition can increase the risk of other diseases, such as type 2 diabetes, cardiovascular disease, hypertension, dyslipidemia, fatty liver, obstructive sleep apnea, musculoskeletal disorders or certain types of cancer, many of which reduce life expectancy [1]. The fundamental cause of obesity is a positive energy imbalance such that energy intake exceeds energy expenditure (energy consumed by the body to maintain life and to perform physical activity). This energy balance is predominantly coordinated by the central nervous system (CNS), which receives neural and chemical input regarding the body’s energy status from the peripheral organs, integrates them and generates a proper response to preserve homeostasis [2]. Some of those signals consist of adipocytokines that are produced by the adipose tissue. Thus, in addition to simply serving as an energetic reservoir, the adipose tissue is also an active endocrine organ that regulates many metabolic processes [3]. Leptin is one of those adipocytokines, and its levels are linked with the nutritional status and energy storage. Consequently, leptin acts as an energy level signal, interacting with the nervous and immune system to trigger responses that regulate body weight and energy homeostasis. Ultimately, leptin reduces food intake and body weight [4]; a lack of leptin signaling in humans and rodents, either due to mutation in leptin or its receptor, leads to an increase in food intake, reduction in energy expenditure and other neuroendocrine problems, such as hypothyroidism, infertility and decreased growth [5,6]. However, abnormal or excessive fat accumulation in the context of obesity can lead to increased leptin levels, producing a phenomenon called “leptin resistance”, in which leptin signaling is attenuated. So, during this physiological resistance, leptin loses the capacity to depress feeding, increase energy expenditure and decrease body weight/adiposity [7]. Several mechanisms have been proposed to explain resistance to the catabolic effects of leptin: alterations in leptin receptor expression, problems with transport of leptin across the blood–brain barrier and/or alterations in development programming [8,9,10].

Leptin is produced primarily by the adipocytes in white adipose tissue (WAT), where it plays important physiological roles both indirectly (primarily via the nervous system) and directly (in an autocrine action). In recent years, we have accepted the adipose tissue as an endocrine organ and adipocytes, along with other constitutive cells, as important regulators of whole-body homeostasis. WAT is capable of expanding or altering adipokine production and release, which serves as a powerful crosstalk between different tissues to modulate metabolic homeostasis. Several processes occur in adipose tissue: when an excess of lipids necessitates WAT expansion, the number or size of adipocytes can increase in a phenomenon called adipogenesis; in contrast, through an apoptosis process, fat loss can occur via reduction of the size or number of adipocytes. During fasting or exercise, lipids stored in adipocytes are mobilized to provide energy to the body. Also, in specific circumstances, white adipocytes are able to burn fat in a process called browning, which has drawn interest as a new possibility for obesity treatment. Finally, WAT is also composed of other cells, such as immune cells, and it is strongly linked to the immune system. Leptin contributes to the regulation of all these processes.

## 2. The Beginning of the Journey: White Adipose Tissue

Adipose tissue is one of the largest endocrine organs in the body; it is involved in a large number of physiological processes. There are two types of adipose tissue: WAT and brown adipose tissue (BAT), which exhibit differences in morphology and functions. Brown adipocytes consist of multiple small lipid droplets packed with a large number of mitochondria. In mammals, BAT is a thermogenic tissue that produces heat to burn fat, and is responsible for adaptative, non-shivering thermogenesis [11]. This unique capacity is attributed to the high number of mitochondria and the expression of uncoupling protein 1 (UCP1) in the inner mitochondrial membrane. When activated, this proton channel protein enables the transport of protons across the membrane, transforming chemical energy (fatty acids) into heat (thermogenesis) [12,13]. BAT is especially important in newborns and hibernating mammals to maintain body temperature and survive cold temperatures. In humans, BAT was primarily considered in newborns and infants, as it was generally accepted that this tissue disappeared or lost functional relevance in adulthood. However, studies have more recently demonstrated BAT existence in adult humans [14], as well as UCP1 expression [15,16]. Since then, countless studies have revealed its functional metabolic role and the implications of this tissue in energy homeostasis and obesity [17,18,19]. In rodents and humans, BAT depots are mainly distributed in the interscapular, axillary, cervical and perirenal areas [20,21], and thermogenic activity can be stimulated in response to cold exposure or noradrenergic stimulation [22].

On the other hand, WAT was long considered an inert tissue to store energy in the form of fat to be mobilized during periods of food deprivation, as well as to provide thermal insulation and mechanical protection [23,24]. White adipocytes contain a single large lipid droplet (90% of the cell volume) with a small number of mitochondria. Anatomically, WAT is widely distributed in different depots; broadly, these depots can be divided into intra-abdominal or visceral (around organs: mesenteric, perigonadal, omental) and subcutaneous (under the skin) compartments [25]. Additional endocrine functions have been attributed to it after noticing that WAT is capable of producing a large number of compounds that can regulate energy balance and other physiological processes. This change was made possible by the discovery of leptin in the ‘90s [26], and, little by little, additional WAT-derived active molecules have been discovered. These active peptides or proteins, called adipocytokines, have actions in a huge number of physiological processes, such as energy metabolism, vascular hemostasis, angiogenesis, immunity and inflammation [27,28]. WAT-derived peptides include leptin, adiponectin, vesfatin and resistin, as well as some pro-inflammatory adipocytokines such as tumor necrosis factor-α (TNF-α), interleukin-6 (IL-6), transforming growth factor-β (TGF-β) and plasminogen activator inhibitor-1 (PAI-1) [29,30]. Depending on its location, different WAT depots exhibit diverse cellular composition, the capacity to secret various adipocytokines, and different innervation and vascularization. This differential distribution of adipose tissue is also related to specific functions. Subcutaneous adipose tissue is implicated in temperature regulation and isolation as well as in sexually dimorphic body compositions. Visceral adipose tissue is located around the organs to serve a structural function [31].

Recently, a third type of adipocyte located in WAT was reported that exhibits characteristics associated with both white and brown adipocytes. They are called beige or brite (brown-in-white) adipocytes and most are located in subcutaneous fat depots [32]. The origin of beige adipocytes has not yet been fully elucidated. In normal conditions, beige adipocytes function as white adipocytes. However, when WAT is stimulated, such as by cold exposure, they can acquire a brown-like phenotype (termed “browning”), evinced by expression of thermogenic genes such as UCP1 and PR domain containing 16 (PRDM16), although the genetic expression patterns are distinct from WAT and BAT [32,33]. Indeed, PRDM16 is necessary for maintaining the beige phenotype, even though UCP1 undoubtedly facilitates thermogenesis [34,35]. Furthermore, adrenergic stimulation plays an essential role in triggering browning, which is facilitated by adjusted sympathetic tone to WAT originating in the brain. In this sense, several neuronal populations in the hypothalamus (the primary regulator of the autonomic nervous system) have been implicated in the control of browning [36,37,38]. Other factors implicated in the activation of browning are immune cells [39,40,41], the metabolic regulator fibroblast growth factor 21 (FGF21) [42], thyroid hormones [43], bone morphogenetic proteins (BMPs) [44,45], nicotine [46,47], cold exposure [48,49] and fasting [50,51]. Leptin also affects browning and will be discussed below.

## 3. Leptin Production

Leptin is a hormone that was identified in mice in 1994 [26] and in humans in 1995 [52], as a product of the *ob* gene. Its function was described as a protector against obesity because the *ob/ob* mice (leptin-deficient) and *db/db* mice (leptin-resistant) were obese, among other signs and symptoms. However, the literature has shown that leptin can also modulate the neuroendocrine axes, autonomic nervous system, neural plasticity and memory. Leptin is produced and secreted primarily from adipose tissue into circulation to have effects in the CNS and peripheral organs. Relatively low levels of leptin are produced by other tissues, such as skeletal muscle, brain, stomach, pituitary gland, mammary epithelium or placenta, but this appears to result in local, as opposed to systemic, actions [53]. Circulating leptin concentration is directly related to adipose tissue size; this informs the brain about available energy storage [54,55]. More leptin is released by subcutaneous relative to visceral depots, and plasma levels are higher in females than males [56].

**Figure 1:**
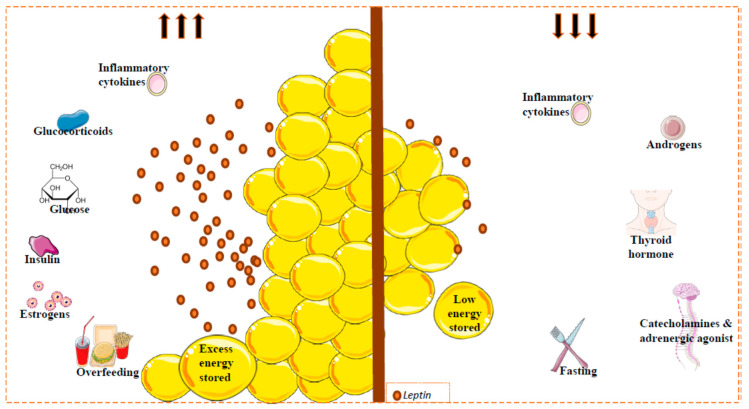
Factors implicated in circulating leptin levels. Leptin is secreted mainly by white adipose tissue. Leptin levels are correlated with the amount of fat (reflecting the amount of energy stored), and with changes in caloric intake. Other factors can regulate the circulating leptin levels. Glucose, insulin, estrogens and some inflammatory cytokines (acute effect) promote leptin secretion (left); catecholamines or adrenergic agonists, thyroid hormones, androgens and inflammatory cytokines inhibit the secretion of this hormone (right). Fat mass and leptin levels are significantly affected by gender and by menopausal status, where the leptin levels can decrease; leptin levels in females are higher than in males. This sexual dimorphism is due, at least in part, to the suppressive effect of androgens on leptin [78].

Leptin expression and circulating levels change with nutritional state, but also display a circadian oscillation with higher values in the evening and early morning [57,58,59]. Consistent with this, during a fasting period or weight loss, leptin levels decrease, whereas during feeding or in obese conditions, levels increase [60]. However, several metabolic and endocrine factors can also regulate leptin expression and secretion. Importantly, in relation to reduced leptin levels, the adrenergic system plays a clear role. Thus, activation of beta 3 adrenergic receptor (β3-AR) (strongly implicated in the regulation of energy balance) by agonists suppresses leptin levels via a pathway downstream of leptin [61,62]. In the same line, cold exposure is accompanied by a reduction in plasma leptin levels [63,64]. Certain hormones, such as growth hormone, thyroid hormone and androgens, appear to reduce leptin levels [65], whereas others, such as estrogens, can increase leptin production in rats and humans [66]. Furthermore, some drugs can also affect leptin production; for example, glucocorticosteroid injection induces adipose tissue *ob* gene expression followed by a decrease in body weight gain and food consumption [67,68,69]. Some proinflammatory cytokines might modify circulating leptin levels incrementally initially, followed by a long-term decrement [70] (Figure 1).

It is important to mention the directly proportional relationship that exists between insulin and leptin levels. Physiological insulin infusion maintains or increases plasma leptin concentrations [71], although acute hyperinsulinemia seems have no effect [72,73]. The direct role of insulin in leptin production is supported by some in vitro and in vivo studies, though some discrepant results have been reported. In vitro, subcutaneous human adipose tissue explants incubated with insulin released more leptin compared with controls, but changes in leptin mRNA levels were not detected [74]. Nevertheless, in cultures of human adipocytes, it seems that insulin is essential for maintaining leptin mRNA levels [75]. When the direct effect of insulin was compared in explants and isolated fat cells in parallel, inhibited leptin secretion was similar in both preparations [76]. In vivo, experiments in mice have shown that levels of leptin mRNA were increased after a single insulin injection in fasted animals [57]. Nutritional regulation of leptin is directly linked to insulin levels; with feeding, when insulin levels are increased, leptin levels increase too. Insulin and leptin are important molecules which are implicated in metabolic homeostasis, and their interactions are of clear interest because of the link with obesity and diabetes. It seems clear that *ob* gene expression is under hormonal control. However, it is a complex scenario to study, as other factors and hormones may be implicated. Also, experimental conditions such as animal, sex, fat depot origin or incubation time [77] could affect the conclusions.

## 4. Leptin Receptor and Signaling

Leptin receptors (LepRs) are encoded by the *db* gene, and are expressed in many tissues. Six LepR isoforms (short and long forms) with different physiological roles have been identified [79]. The long isoform (LepRb) has an extracellular N-terminus ligand-binding domain (similar to other isoforms), a transmembrane domain and a complete intracellular domain required for the intracellular signaling pathways. This fully active isoform is essential for leptin action and is expressed in the CNS, mainly in the hypothalamus [80,81,82]. LepRb is implicated in energy homeostasis; in this sense, the *db/db* mice (which lack LepRb) have an obese phenotype, with diabetes, pubertal disorders and elevated leptin levels [83]. The majority of these receptors are localized inside the cell, and the main signaling pathway recruited by leptin is the JAK2/STAT3 pathway. When leptin binds to LepRb, the receptor changes its conformation and Janus quinasa 2 (JAK2), after translocation, becomes activated by autophosphorylation and activates specific tyrosine residues (Tyr985 and Tyr1138) in the receptor. Tyr1138 phosphorylation allows binding to occur of Signal Transducer and Activator of Transcription 3 (STAT3) (a transcription factor) to an intracellular motif. Subsequently, STAT3 migrates to the nucleus and transcriptionally regulates expression of specific genes [82,84]. Tyr985 is a binding site for the suppressor of cytokine signaling 3 (SOCS3) that exerts inhibitory effects on leptin signaling via a negative feedback loop [85]. Also, phosphorylation of another tyrosine residue, Tyr1077, recruits STAT5 and contributes to the leptin action in energy balance and reproduction [86,87]. The phosphatidylinositol 3-kinase (PI3K) pathway can be activated by leptin too. In peripheral tissues and hypothalamus, this pathway is implicated mainly in insulin actions [88], but leptin can activate PI3K and phosphodiesterase 3B (PDE3B) and provoke a reduction in cAMP levels. This PI3K-PDE3B-cAMP pathway interacts with JAK2-STAT3 pathway and constitutes another essential component of leptin signaling [89,90].

Regarding the short isoforms, LepRe is a soluble isoform that acts as a leptin-binding protein. This receptor can neutralize leptin, removing it from circulation to regulate energy homeostasis [91]. LepRa is expressed in kidney, lung, choroid plexus and in cerebral microvessels [81,92]. This short isoform may play a significant role in the transport of leptin through the blood–brain-barrier (BBB) to the brain, and can contribute to the actions of leptin in obesity [93]. Although LepRb is highly expressed in brain, specifically in areas linked to metabolic regulation (arcuate nucleus, dorsomedial nucleus and ventromedial nucleus), both long and short isoforms have been described in various peripheral tissues such as ovary, uterus, testis, lung, kidney, liver, adrenal gland, placenta, peripheral blood mononuclear cells, WAT and BAT [79,81,94,95,96]. They may be target sites for leptin actions, although their expression is lower compared with that of the CNS, especially the long isoform [95]. However, LepR isoforms have been demonstrated in adipose tissue, including the full-length isoform [97,98,99]; in the adipocyte, LepR is located on the cell membrane and in small cytoplasmic vesicles [100]. A few years after the discovery of leptin, its direct actions were confirmed; leptin treatment of white adipocytes in vitro induced activation of the JAK/STAT pathway and increased expression of target genes, suggesting the activation of the mechanism of leptin action through its receptor [101]. LepR is expressed by adipose tissue-resident immune cells and endothelial cells [100]. This suggests not only a central action, but also an autocrine and paracrine action for leptin.

## 5. Leptin and Adipogenesis

Adipose tissue is comprised of different cells types. Adipocytes constitute a third of the tissue and the remaining cells are preadipocytes, fibroblasts, stromal cells, T-cells, granulocytes, macrophages and monocytes [31]. The differentiation of preadipocytes to adipocytes (adipogenesis) depends on energy status; it is controlled by hormonal activity and transcription factors [102], and this capacity declines with age [103]. Adipose tissue has the ability to grow when the capacity to store lipids is insufficient. Thus, the growth can be by hyperplasia (increasing the cell number) and hypertrophy (increasing the cell size) [104]. Although one or both types of growth can occur, hyperplasia primarily occurs in early growth states, and hypertrophy occurs previous to hyperplasia during the progression of obesity in most depots [104,105]. However, in humans, these processes are still not completely understood. Depending on the fat depots, different growth patterns can occur. In obese women, it was reported that hyperplasia is predominant in the subcutaneous fat pad, and that hypertrophy occurs both in the omental and subcutaneous depots [106]. In adult humans, the increased lipid storage in adipocytes seems to be the most important process to accumulate fat mass [107,108]. Recently, Spalding et al. observed that the number of fat cells remains constant in lean and obese individuals, and that the number of adipocytes seems to be established at an early age [109]. Although adipogenesis in adult declines, it can still occur; this may be due, in part, to the expression of peroxisome proliferator-activated receptor γ2 (PPARγ2). This receptor is a key regulator of adipogenesis [110], and is more highly expressed in younger relative to older people [111].

Hyperplasia and hypertrophy in adipocytes occur in a leptin-rich environment, so it is interesting to consider the possibility that leptin could exert some paracrine or autocrine effects on adipocyte differentiation. Functional LepRa and LepRb were identified in subcutaneous preadipocytes [112]. In response to a physiological leptin dose, mitogen-activated protein kinase (MAPK) and STAT pathways were activated in rat preadipocytes, promoting adipogenesis. Also, an increase in PPARγ2 expression, lipoprotein lipase levels and more fat storage was observed in these cells [112]. However, direct exposure to high concentrations of leptin (250 and 500 ng leptin/mL) could inhibit proliferation [113]. In the same study, it was observed that leptin could indirectly inhibit the proliferation of preadipocytes by activation or inhibition of unidentified circulating factor(s) [113]. In contrast, in pigs, this inhibition was not observed; thus, leptin in porcine preadipocytes proliferation, even with high dose (1000 ng/mL)- stimulated proliferation [114]. If we compare the results from primary culture with results from 3T3-L1 cells (line derived from murine fibroblasts), some controversial data emerge. Leptin (at dose 5 to 500 ng/mL) had no effect on 3T3-L1 proliferation but suppressed lipid accumulation and increased glycerol released during the maturation process of these cells [115].

Recent studies in vitro in 3T3-L1 cells have shown that leptin (in a similar concentration to the circulating levels of leptin in obese individuals) enhances the expression of adipogenesis- and lipogenesis-related factors (included PPARγ). It also promotes the formation of lipid droplets in preadipocytes by the mTOR signaling pathway [116]. The variations in in vitro studies that have focused on the actions of leptin in this process could be explained by the various sources of leptin and the origin of the preadipocytes; nonetheless, it seems that under experimental conditions, high leptin levels could promote the differentiation of adipocytes. In overweight individuals, leptin levels are high, and an increase in preadipocyte number to facilitate the expansion of fat depots could be expected to avoid fat accumulation in other tissues. However, in natural conditions, i.e., in a healthy animal, leptin production decreases in parallel with the decrease in fat depots.

## 6. Leptin and Apoptosis

For the purpose of maintaining homeostasis, cells can suffer apoptosis, a normal phenomenon of cell death. Apoptosis is characterized by the activation of caspases (protease enzymes with essential role in programmed cell death); these proteases play a critical role in the execution phase of this process, and their actions lead to cell death via digestion of genomic DNA into oligonucleosomal fragments, among others. DNA fragmentation is considered a symbol of apoptosis [117]. Fat loss could be achieved by decreasing the number of adipocytes through apoptosis. Endogenous elimination of adipocytes has been observed in rats with streptozotocin-induced diabetes, in humans with malignancy and in humans with lipodystrophy [118,119,120]. It seems that adipose tissue apoptosis is linked to various conditions associated with weight loss.

The link between leptin and apoptosis has received much attention since this hormone was discovered. Early reports showed that leptin could modulate this process via CNS. The adipose tissue of leptin-intracerobroventricular (ICV) treated rats showed characteristics particular of apoptosis and a reduction in WAT mass [121]. At the intracellular level, leptin produced a significant increase in PPARγ levels, but a decrease in TNFα, probably a secondary effect of reduced fat depots [121,122]. It has been postulated that PPARγ is a mediator of leptin-induced apoptosis. However, leptin peripheral administration in mice resulted in big decreases in fat pad size, but without the presence of apoptotic markers [123]. Something similar happened with rats made hyperleptinemic by hepatic overexpression after adenoviral gene transfer, i.e., the size of the fat decreased but without changes in the DNA content and with any important decrease in PPARγ [124]. These discrepancies could be explained by the different routes of leptin administration.

Leptin supplementation in adipocytes culture induced the expression of angiopoietin-2 (Ang-2), antagonist of angiogenesis, and apoptosis in the endothelial cells [125]. A decrease in endothelial proliferation could be associated with adipose apoptosis [126]. Leptin can increase reactive oxygen species (ROS) in endothelial cells [127,128], and these species are involved in the induction of apoptosis [129], showing another possible connection. Leptin-induced adipose tissue apoptosis was also studied in *ob/ob* mice. Della-Fera et al. injected different leptin doses over 14 days in *ob/ob* and *ob*/? mice; the first group appeared to be more sensitive than the latter to leptin-induced adipose tissue apoptosis, but there were differences between fat depots [130].

The specific mechanisms that control the apoptotic leptin actions are not clear. However, a study showed that after blocking the neuropeptide Y (NPY) receptor, the adipose tissue apoptosis increased [131]; it is known that leptin provokes a reduction in hypothalamic NPY levels [132], so together, these factors could suggest a link between NPY and the leptin-induced adipose apoptosis. Other reports have provided the opposite idea, i.e., an anti-apoptotic role for leptin, but they were done in brown adipocytes, which is not going to be discussed here [133,134,135]. Over the years, adipocytes were considered very stable, so leptin-induced adipocyte apoptosis is a relatively new concept that needs to be explored in more depth.

## 7. Leptin and Lipolysis

Energy is stored mainly as triglycerides (TAG) in WAT. The process to mobilize this reservoir is known as lipolysis, i.e., sequential hydrolysis that breaks the TAG to release glycerol and non-esterified fatty acids. The first step is catalyzed by adipose triglyceride lipase (ATGL) that hydrolyzes TAG to diacylglycerol (DAG) and one fatty acid. Afterwards, DAG is transformed to monoacylglycerol (MAG) and another fatty acid by a different enzyme called hormone-sensitive lipase (HSL). Finally, by the enzyme monoacyglycerol lipase (MGL) actions, MAG is hydrolyzed to glycerol and an additional fatty acid (FA) [136,137]. When energy is required, the utilization of FAs is not the same in all fat depots; subcutaneous and some visceral depots (retroperitoneal and mesenteric) are the first to be mobilized [138]. Lipolysis is a vital process, not only providing energy for other tissues, but its intermediate and final products can act as signaling molecules to regulate several metabolic processes. This process happens under basal conditions (non-stimulated) or in conditions stimulated by hormones. For example, under different stimuli, such as periods of fasting or high energy demand, adipocytes are stimulated by hormones (primarily catecholamines) and generate intracellular reactions that activate the enzymes necessary to accelerate lipolysis [139,140]. Adrenergic receptors are widely distributed on the surface of adipocytes in WAT, indicating that the sympathetic nervous system (SNS) plays an important role regulating lipolysis. Also, the contribution of the adrenal gland to this regulation is subdominant, since the adrenodemedullation in rats does not stop lipolysis; thus, catecholaminergic stimulation is transmitted through the SNS to these adrenergic receptors in adipocytes [141]. Anatomic SNS outflow from the CNS to WAT was demonstrated using a viral transsynaptic retrograde tract trace [142]. This bidirectional connection is essential for lipid mobilization, and also has implications in the fat cell number [143,144,145,146]. Leptin exerts actions on sympathetic nerve activity that can affect appetite, energy expenditure and arterial pressure [147]. Regarding the effects in the sympathetic tone to WAT, there are several studies showing its impact. Almost 20 years ago, Sahu et al. showed in rats that, after central chronic leptin infusion, the epididymal fat weight decreased drastically without variations in food intake [148]. Later, another study with leptin infusion into the medio-basal hypothalamus (MBH) showed a suppression in the epidydimal WAT lipogenesis independently of STAT3 signaling, but where an intact autonomic innervation was required [149]. Also, peripheral administration of leptin can excite the sympathetic tone innervating WAT, regulating the lipolytic process specifically in the inguinal fat depots. The authors of this study specifically postulated that leptin travels to the brain through the BBB, and via central histamine H1 receptors, the sympathetic outflow to the WAT is triggered [150].

However, there have been some contradictory findings that are worth mentioning. Rooks et al. revealed interesting results in sympathectomized mice in just one epididymal fat pad by local injection of 6-hydroxydopamine (6OHDA). Explicitly, they observed a reduction in both fat pads, i.e., the intact and denervated, after a peripheral leptin infusion, suggesting that the SNS was not essential for leptin to decrease fat mass and the possibility of an existing communication between fat depots [151]. Similarly, the results of Penn et al. supported the same idea: leptin could reduce the fat size independent of the SNS [152]. In one of the most recent and revealing works, Zeng et al. [153] reconstructed a 3D anatomical picture of the entire subcutaneous fat pad in mouse, revealing a thick bundle of sympathetic neuronal projections in close proximity to adipocytes and in contact with some of them. This was possible using optical projection tomography together with multiphoton microscopy. The authors also showed that the optogenetic activation of these axons increased the levels of noradrenaline, activated HSL, and reduced fat mass. Interestingly, leptin actions in HSL were blocked after disrupting neuronal input surgically, genetically or pharmacologically, signifying that this local innervation is essential for the lipolysis leptin effects [153].Very recently, an important paper was published regarding the mechanisms involved in the effects of leptin on thermogenesis and lipolysis [154]. The effects of acute and chronic leptin treatment on SNS innervation, the main regulator of lipolysis, were evaluated. Low sympathetic innervation in WAT from *ob/ob* mice was ameliorated (anatomically and functionally) following peripheral chronic leptin treatment. They showed that Brain-Derived Neurotrophic Factor (BDNF)-expressing neurons in the paraventricular nucleus were downstream of agouti-related peptide (AGRP) and proopiomelanocortin (POMC) neurons in the arcuate nucleus of the hypothalamus (ARC), and are essential for the leptin actions restoring sympathetic innervation of WAT in *ob/ob* mice [154]. These findings also demonstrate that leptin regulates the architecture of the SNS in the adipose tissue.

Leptin receptor is present in adipocytes, so the study of the possible effect of this hormone directly in lipolysis, in an autocrine/paracrine action, began years ago. In vitro, leptin treatment of isolated adipocytes (pooled from different fat depots of lean mice or *ob/ob* mice) produced an increase in lipolytic activity [155]. Similar results were found in rat epididymal WAT explants [156] and in subcutaneous and perirenal adipocytes [101,157]. Nevertheless, in human adipocytes, this direct lipolytic effect was not observed in adipocytes from children or adults [158]. Also, obese Zucker rats and *db/db* mice (both possess a mutation in the leptin receptor) do not respond to leptin-induced lipolysis, so seems that the leptin receptor is indispensable for the lipolytic actions [101,159]. Most recently, Pereira et al. observed that some enzymes involved in lipolysis and lipid synthesis decreased in mice with specific deletion of LepR in perigonadal WAT, however they did not find difference in adipose tissue lipolysis [160]; also, excision of LepR specifically in adipose tissue slightly reduced body weight, suggesting that this specific LepR knockdown does not affect lipid turnover [160]. This contrast with the results obtained by Huan et al., who used antisense RNA to downregulate LepR expression in WAT, and observed a strong dysregulation in fat metabolism [161]. The different strategies used could explain the differences; notably, the first study targeted just the long isoform in both BAT and WAT, while the second targeted all isoforms only in WAT. All together, these data suggest that leptin may play roles not only in an endocrine or hypothalamic fashion, but also through an autocrine or paracrine pathway. However, some questions still need be answered, for example, the exact mechanism triggering lipid mobilization after sympathetic denervation is not clarified. Also, leptin acts in several brain areas, but the neuronal networks implicated in the regulation of lipolysis or the control of these sympathetic branches in the fat mass are still unclear.

## 8. Leptin and Browning

In the last years, the browning (or *beiging*) of WAT has continuously generated interest because of the potential to increase energy expenditure. Beige adipocytes, together with brown adipocytes, have been linked to obesity resistance in many mouse models [162,163,164], suggesting that beige adipocytes could serve as a therapeutic target to treat metabolic disease. Nevertheless, the thermogenic potential and contribution of this browning process to energy homeostasis is still controversial [165]. The predominant idea was that UCP1 was expressed exclusively in BAT, but lately, some reports showed expression of this molecule in WAT and even in skeletal muscle [166]. Commins et al. showed that peripheral leptin treatment of *ob/ob* mice induced UCP1 expression in WAT, and this result was considered indirect evidence of increased sympathetic nerve activity (SNA) [167,168]. Using a volume fluorescence-imaging technique, dense sympathetic arborizations were detected in mouse inguinal WAT, showing that these sympathetic fibers originated from celiac ganglia, were in close apposition to most of the adipocytes and were activated by cold exposure [169]. One year later, these observations were supported by 3D whole-tissue technique showing WAT architecture and the dense sympathetic innervation on it [170]. The role of this intra-adipose sympathetic arborization seems to be critical for the cold-induced browning, but it is not clear whether they could control the production of hormones such as leptin [169,171]. However, fasting and chemical-genetic activation of AgRP neurons in hypothalamus suppress the adipocyte browning, specifically in retroperitoneal and inguinal WAT; after activation of these orexigenic neurons, browning was suppressed by regulating sympathetic activity [36]. On the other hand, leptin production is altered by fasting and cold exposure too, suffering a reduction in *ob* gene expression followed by a decrease in its blood circulating levels [172,173,174]. Specifically, it was observed that in mice with overnight cold exposure, a clear stimulus for *beiging*, there was a suppression of *ob* gene expression in WAT and this was mediated by the SNS [63]. It is clear that the SNS is implicated in browning and also in leptin secretion, and both are influenced by different stimuli with diverse responses, but the pathways involved in leptin actions on *beiging* are not completely clear. In the last years, some new discoveries provided more information about the mechanisms involved in its actions. In 2007, Plum et al. established that PI3K activation in specific leptin-responsive neurons of the CNS regulated sympathetic nerve activity in WAT, leading to elevated mitochondria content and UCP1 expression. This had an impact on energy homeostasis with an increased energy expenditure and leanness [175]. Years later, some authors suggested that browning could be a combined melanocortin response between leptin and insulin and both were required for optimal CNS-mediated WAT browning. Specifically, they showed that the coinfusion of both hormones into the CNS promoted WAT browning acting through hypothalamic POMC neurons, and they were essential for the optimal CNS-meditated WAT browning. In this complementary leptin and insulin signaling action, two phosphatases expressed in ARC POMC neurons, T cell protein tyrosine phosphatase (TCPTP) and protein tyrosine phosphatase1B (PTP1B), seem to be involved [37]. Other proteins, as Forkhead box C2 protein (Foxc2) with important roles in the regulation of several cellular processes included energy metabolism, were linked to the leptin signal and the browning process. Thus, Foxc2 increases the activity of JAK2/STAT3 pathway [176]) leading to increased browning activity in WAT by elevating the expression of thermogenic proteins as PRDM16, UCP1 and PGC1α, but also increasing leptin transcription [177]. An indirect negative effect of leptin in browning has been shown recently. In muscle, the myokine fibronectin type III domain-containing five (FNDC5) after a cleavage process, release a soluble protein called irisin, that acts on white adipose cells to increase UCP1 expression and browning in WAT [178]. When leptin is administrated in mice, the Fndc5 transcription is downregulated in subcutaneous adipocytes, and the irisin-stimulated expression of Ucp1 and Cidec is also reduced in this tissue, although its administration increases the FNDC5 expression in muscle favoring the myogenesis [179]. This suggests a cross-talk between WAT and skeletal muscle where leptin could have a role. The Hedgehog (Hh) signaling pathway is a signal transduction pathway in several biological process and its abnormal activation can result in tumor development [180]. Recently some authors reported with in vitro studies that leptin can interact with this pathway, upregulating the expression of Gli (the key transcription factor in Hh signaling pathway) and in consequence, inhibiting the adipogenesis process [181]. However, another study showed that leptin could inhibit the Hh pathway and promote adipocyte browning [182]. Nevertheless, the specific effect and the mechanism involved should be explored in the future. As previously mentioned, leptin can also exert autocrine actions, and its receptor is expressed in adipose tissue. It does not appear that papers describing a direct/autocrine role for leptin in the browning process are not yet published. However, some papers have shown leptin effect in browning in in vitro experiments, so an autocrine effect seems that exist, but its contribution to and implication for full body homeostasis have yet to be elucidated.

## 9. Leptin and Inflammation

As we previously discussed, adipose tissue is a dynamic endocrine organ that produces and secretes adipokines as well as pro- and anti-inflammatory cytokines that play an important role in body homeostasis. In obesity conditions, chronic low-grade inflammation is developed and the circulating level of pro-inflammatory cytokines are increased. In this status, WAT is the major source of inflammation associated with obesity [183,184]; the adipose depots suffer an expansion and this is accompanied by the accumulation of immune cells, including infiltrated macrophages with specific characteristics [185]. Leptin, of which levels are increased during obesity, regulates the inflammatory response at several levels (development, proliferation, maturation and activation) and fundamentally, it stimulates the production of pro-inflammatory cytokines [116,186]. Also, most of the immune cells express LepR [187]. All together, these data lead us to consider leptin as a connector among the neuroendocrine and immune system [188,189]. Some reports already have described interesting actions of leptin directly in adipose tissue macrophages (ATM), the most abundant inflammatory cells in WAT. ATMs exhibit an M2 cell phenotype (alternatively activated macrophages; anti-inflammatory type) but they produce cytokines and chemokines usually produced by M1 cells (classically activated macrophages; pro-inflammatory type). Recently, Acedo et al. investigated the role of leptin in the polarization process of macrophages found in adipose tissue. After a leptin exposition, M2-phenotype markers were found in these cells, but also the capacity to produce pro-inflammatory cytokines (M1) [190]. As in previous data, they found the same specific ATM phenotype, but leptin could have a role in their polarization. However, one year later in an interesting study in vivo, other authors have shown that leptin can stimulate the cAMP signaling pathway via the histone deacetylase HDAC4 in ATM, producing a decrease in inflammatory gene expression [191]. Leptin actions in resident macrophages can also be indirectly through mast cells, a migrant cell of connective tissue that contribute to obesity and diabetes [192]. Leptin expression in mast cells from diet-induced obesity (DIO) WAT is higher than those from lean WAT.

Interestingly, leptin-deficient mast cells drive macrophage polarization from M1 towards M2 phenotypes, however do not affect T-cell differentiation [193]. *Ob/ob* mast cell (leptin-deficient mast cells) were transferred to WT mice and the results observed were surprising: reduction in body weight gain, WAT weight and adipocyte size [193]. Furthermore, the transfer of *db/db* bone marrow to WT mice placed on high fat diet reduced macrophage infiltration and adipose tissue inflammation with a reduction in adiposity [194]. Leptin has been shown to moderate obesity-related inflammation through mast cells. Some evidences in human adipocytes support the idea that leptin could initiate the recruitment of macrophages to adipose tissue. Leptin could provoke endothelial cell activation and start the recruitment of circulating blood monocytes into fat pads, but this action could be not exclusive since other adipokines could participate too [195]. One of the most recent studies which was already mentioned in previous sections, links leptin inflammation actions with Foxc2; this specific protein is also implicated in the inflammatory process, alleviating it into the adipocyte by downregulation of leptin-JAK2/STAT3-PRDM16 pathway [177]. Despite these findings, some data also support the idea that leptin has no effects in macrophage infiltration in the adipose tissue. For example, Gutierrez et al. observed that hematopoietic LepR deficiency does not affect macrophage accumulation [196] and Gove et al. showed that LepR signaling in bone marrow cells is not implicated in WAT inflammation [197]. Although it is clear that infiltrating macrophages are increased in WAT in models of genetic or high-fat-diet induced obesity and they play an active role in this pathology, mice that are leptin or LepR deficient (*ob/ob* and db/db respectively) present lower levels of these macrophages and low inflammatory gene expression, even with higher body weight and adiposity [198,199]. Existence of leptin actions in the immune system are clear, but more contributions regarding its role in adipose tissue inflammation, macrophages recruitment and the specific pathways involved are warranted in this novel and fascinating topic.

## 10. Conclusions and Future Therapeutic Perspectives

The identification of leptin and its crucial role as a central regulator in energy metabolism has opened a new door to increase our knowledge in the factors implicated in obesity and its associated metabolic disorders. Leptin gene mutations in humans are rare [200], and the treatment of obesity with leptin is only effective in a minority of individuals [201], suggesting that leptin may not be the most promising approach for obesity treatment. This effect is, in part, because of the leptin resistance observed in obese individuals, where the exogenous leptin treatment has diffident effects. For this reason, leptin resistance could be considered one of the most important risk factors for overweightness and obesity. In order to find effective treatments against obesity and its comorbidities, it is important to better understand this leptin resistance phenomenon and the underlying mechanisms. Problems in the LepR signaling pathway are predictable causations of leptin resistance; for example, a decrease in LepR trafficking to the cell surface [202,203] and/or increase in the internalization of the receptor via endocytosis [204] could contribute to leptin resistance due to a reduction in plasma membrane LepR. Also, alterations in intracellular proteins implicated in leptin signaling, such as SOCS3 [205], PTP1B and TCPTP [206,207], or Src homology 2B 1 (SH2B1) [208], may contribute to this phenomenon too. Leptin acts mainly through the CNS, so modifications in the hypothalamic neural circuitry could also contribute to the leptin resistance. Thus, inhibition of a BDNF pathway via melanocortin 4 receptor (MC4R)-dependent mechanism in ventromedial nucleus of the hypothalamus (VMH) results in leptin resistance, along with hyperphagia and obesity [209,210]. Recently, it was shown that overexpression of Pblorotein tyrosine phosphatase receptor type J (Ptprj), expressed in hypothalamic neurons together with leptin receptors, could be a factor contributing to the development of leptin resistance [211]. Most of the leptin receptors are in the CNS, and the transport of leptin into the brain is one of the mechanisms that could be implicated in this resistance phenomenon; consequently, this process is damaged with obesity in humans and mice models [9,212]. In humans, Banks et al. found that triglycerides can cross the BBB and induce leptin resistance [213]. Leptin may also exert its activity at a peripheral level, for that reason peripheral leptin resistance can occur in parallel to central leptin resistance.

It is important to mention that some factors can contribute to or cause leptin resistance under an obesity context. Hyperleptinemia itself can cause leptin resistance by downregulating cellular response to leptin [214], thus reversing the hyperleptinemia by reducing leptin expression or secretion by adipocytes could be a approach to control obesity effects [215]. Endoplasmic Reticulum (ER) stress occurs when there is an accumulation of unfolded or misfolded proteins in the ER lumen, and this is associated with several metabolic diseases including obesity [216]. Specifically, obesity cause ER stress in the hypothalamus which can lead to inhibition of leptin receptor signaling contributing to leptin resistance [217,218]. Reducing central ER stress could be an approach to improve leptin resistance and ameliorate obesity problems [219,220,221]. External factors can have implications in leptin resistance too. For example, specific diets with high content in sucrose or salt, or physical activity, can have implications in leptin sensitivity [222,223,224]. Some compounds have shown positive effects reducing leptin resistance; liraglutide (a glucagon-like peptide-1 (GLP1) analogue) reverse this problem by improving endothelial function and reducing inflammatory signals [225]. Chitosan Oligosaccharide Capsules (COSCs) seems to mitigate leptin resistance by activation of JAK2/STAT3 pathway in obese rats [226] and KBH-1 (an herbal mixture) could alleviate this phenomenon by regulation of the AMP-activated protein kinase (AMPK) pathway [227]. Finally, the neuropeptide oxytocin, when administered subcutaneously, showed positive effects reducing the acute but not the chronic leptin resistance in obese mice [228]. Leptin administration in an obesity context has an insignificant impact because of this phenomenon of leptin resistance; for that reason, it is crucial to continue studying the mechanisms underlying this metabolic event.

**Figure 2:**
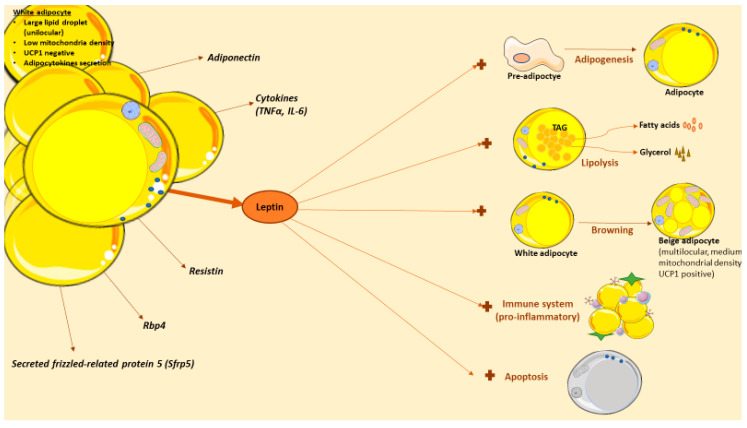
Leptin actions in white adipose tissue. White adipose tissue (WAT) is composed of by adipocytes, among other cells types. White adipocytes are cells with capacity to store lipids in a large droplet. WAT adipose tissue is more than a reservoir, as it also serves as an endocrine organ that produce and release several adipocytokines with effects in obesity, including leptin. Leptin has several effects in WAT, in direct or indirect mechanisms. Thus, leptin can promote adipogenesis, lipolysis or browning in this tissue. Recently, leptin actions in immune system have sparked the interest in the field; briefly, this hormone stimulates the production of pro-inflammatory cytokines and it is implicated in adipose tissue macrophages (ATMs) polarization. Also, leptin stimulates the apoptotic adipocyte process, another promising area since fat loss could be achieved by decreasing the number of adipocytes through apoptosis.

Likewise, leptin actions are also interesting in other pathologies because of the wide number of biological processes that is implicated (Figure 2), especially under energetic deficit. For example, in lipodystrophy, a condition with abnormal or degenerative adipose tissue unable to accumulate fat; this pathology causes lipid accumulation in other organs leading to the development of secondary problems such as insulin resistance, hepatic steatosis or dyslipidemia [229]. Lipodystrophy can be partial (adipose tissue abnormalities in one or more sites) or generalized (near-total lack of body adipose tissue), and congenital or acquired (caused by autoimmune-mediated destruction or iatrogenic-induced dysfunction of adipose tissue) [230]. Deficiency of adipose tissue can occur in different body areas as well as include different amounts of fat loss, both of which determine the levels of hyperleptinemia and insulin resistance [230].In these patients, exogenous leptin administration improves the metabolic problems associated to lipodystrophy [231,232,233,234,235]. However, some lipodystrophic patients cannot be hypoleptinemic, so the treatment can be more challenging. In caloric-restricted circumstances, leptin levels are low and this can lead to problems in the reproductive axis [236], for example, in functional hypothalamic amenorrhea. In this condition, the existence of a chronic negative energy balance because of excessive exercise or low food intake leads to a leptin deficiency [237]. Women with hypothalamic amenorrhea can benefit from leptin treatment; specifically, some studies have shown an improvement in reproductive, thyroid and growth hormone axes in these patients after receiving a 3-month recombinant leptin therapy [238,239]. Moreover, bone mineral density was improved [239,240]. Anorexia nervosa is characterized by refusal to keep body weight and it is also associated with low concentrations of leptin and neuroendocrine abnormalities [241]; in this context of energy deprivation, leptin treatment has shown positive impacts and a protective effect [242,243,244]. Finally, although in type 2 diabetes leptin treatment shows small effects (probably because of the high serum leptin levels), in type 1 diabetes the effects are more promising. Thus, leptin therapy seems to improve levels of hepatic intermediary metabolites, drops lipogenic and cholesterologenic factors, and reduces plasma and tissue lipids [245,246]. Though these studies are in animals, the results suggest a promising therapeutic potential against diabetes.

In other diseases where WAT is altered, leptin has important implications too. In Crohn’s disease where fat-wrapping is one of the main characteristics, leptin has been implicated in the intestinal inflammation associated with these patients; in this way, hyperplastic mesenteric fat, wrapping inflamed intestinal segments, produce leptin and other adipokines that modulate the systemic immune cell composition and the intestinal cell function [247,248]. These patients can suffer at the same time acquired lipodystrophy; in this context, where leptin treatment could have benefits, it is important considerer that also could result in an aggravation of the intestinal inflammation [249]. In the same context, in inflammatory bowel disease (IBD) the mesenteric WAT is hypertrophied; these patients are characterized by suffering anorexia and altered body composition. Leptin levels are also altered in these patients showing overexpression of this adipokine in mesenteric WAT [250]. These data are really important in order to stablish therapeutic interventions for these patients.

WAT is no longer considered an inert reservoir; it can produce a wide number of molecules, as well as include different kind of cells (adipocytes, vascular cells, endothelial cells or immune cells). Leptin is the main adipokine produced by this tissue, and although it has functions in many other tissues, its functions in the same tissue that produce it are essential and have implications in several metabolic processes. Since the discovery of leptin in 1994, countless studies have provided details about its production, functions and pathways, but a better understanding of the precise mechanisms implicated may lead to new approaches and new therapeutic applications. Every year, new studies provide more details or roles about this fascinating hormone, for example the recent leptin role in immunometabolism opening a new window in the field of immunology. Also, leptin resistance is still one of the biggest problems in obesity, where more doubt should be resolved to try to find solutions to one of the biggest problems of the 21 centuries. I strongly believe that, regarding leptin, “*there is much else that may be told*”.
